# The effect of oral acetazolamide on cystoid macular edema in hydroxychloroquine retinopathy: a case report

**DOI:** 10.1186/s12886-017-0517-0

**Published:** 2017-07-12

**Authors:** Eun Hee Hong, Seong Joon Ahn, Han Woong Lim, Byung Ro Lee

**Affiliations:** Department of Ophthalmology, Hanyang University Hospital, Hanyang University College of Medicine, #17 Haengdang-dong, Seongdong-gu, Seoul, 133-792 South Korea

**Keywords:** Acetazolamide, Case report, Cystoid macular edema, Hydroxychloroquine retinopathy

## Abstract

**Background:**

Hydroxychloroquine (HCQ) retinopathy can accompany other retinal complications such as cystoid macular edema (CME), which leads to central visual loss. We report a case of CME with HCQ retinopathy that improved with the use of oral acetazolamide, and discussed the possible mechanisms of CME in HCQ retinopathy using multimodal imaging modalities.

**Case presentation:**

A 62-year-old patient with systemic lupus erythematosus (SLE) and HCQ retinopathy developed bilateral CME with visual decline. Fluorescein angiography (FA) showed fluorescein leakage in the macular and midperipheral area. After treatment with oral acetazolamide (250 mg/day) for one month, CME was completely resolved, best corrected visual acuity (BCVA) improved from 20/50 to 20/25, and FA examination showed decreased dye leakage in the macular and midperipheral areas.

**Conclusions:**

In cases of vision loss in HCQ retinopathy, it is important to consider not only progression of maculopathy, but also development of CME, which can be effectively treated with oral acetazolamide.

## Background

Hydroxychloroquine (HCQ) retinopathy is a form of retinal toxicity caused by HCQ and is characterized classically as a bilateral bull’s-eye maculopathy, in which there is a ring of parafoveal retinal pigment epithelium (RPE) depigmentation with sparing of the fovea. HCQ retinopathy can accompany other retinal complications such as cystoid macular edema (CME) and epiretinal membrane [1]. Specifically, CME is the result of fluid accumulation in the outer plexiform layer in the macular area and leads to central visual loss.

Several treatment modalities have been shown to be effective for CME combined with other retinal diseases. In eyes with combined CME and retinitis pigmentosa, treatment with oral or topical acetazolamide can produce successful resolution of CME and functional improvement according to previous studies [2–7]. Although the pathogenic mechanism of CME in HCQ retinopathy has not been elucidated, oral or topical acetazolamide may also be useful in the treatment of CME in HCQ retinopathy. Here, we report a case of CME in HCQ retinopathy that improved with the use of oral acetazolamide. Using multimodal imaging modalities, we explored the detailed retinal structural changes, and discussed the possible mechanisms of CME in HCQ retinopathy.

## Case presentation

**Fig. 1:**
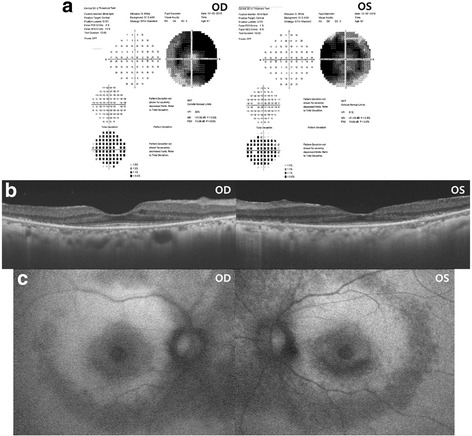
Diagnosis of hydroxychloroquine retinopathy based on visual field examination, optical coherence tomography, and fundus autofluorescence. **a** Humphrey 30-2 visual field test shows dense paracentral ring scotoma with decreased foveal sensitivity in both eyes. **b** Spectral-domain optical coherence tomography (SD-OCT) demonstrates defects in the paracentral photoreceptor layer. **c** Fundus autofluorescence reveals decreased paracentral and mid-peripheral fluorescence in both eyes

A 60-year-old woman visited our clinic complaining of blurred vision in both eyes. The patient had been diagnosed with systemic lupus erythematosus (SLE) and had taken HCQ for the past 20 years. Her daily dose of HCQ was 400 mg, and her total cumulative dose was estimated to be 2920 g. The patient had first come to our clinic 7 months prior, at which time her best corrected visual acuity (BCVA) was 20/30 in both eyes and fundus examination showed bilteral midperipheral retinal degeneration. Spectral-domain optical coherence tomography (SD-OCT) showed defects in the paracentral photoreceptor layers. Consistent with this finding, a visual field test revealed dense paracentral ring scotoma with decreased foveal sensitivity in both eyes (Fig. 1). The patient reported no family history of eye diseases and no visual symptoms before the initiation of HCQ therapy, and she had no auditory symptoms. At that time, the patient was diagnosed with HCQ-induced retinal toxicity, HCQ retinopathy, and HCQ treatment was discontinued. Four months later, BCVA was maintained as 20/30 in both eyes.

**Fig. 2:**
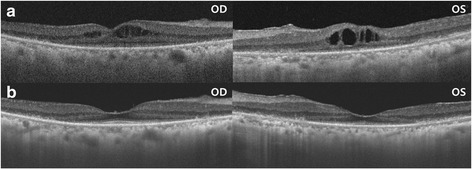
**a** Cystoid macular edema (CME) shown by spectral-domain optical coherence tomography (SD-OCT). **b** The patient’s CME completely resolved after treatment for one month with oral acetazolamide. Central macular thickness (CMT) decreased from 245 to 177 μm and from 335 to 146 μm in the right and left eyes, respectively

**Fig. 3:**
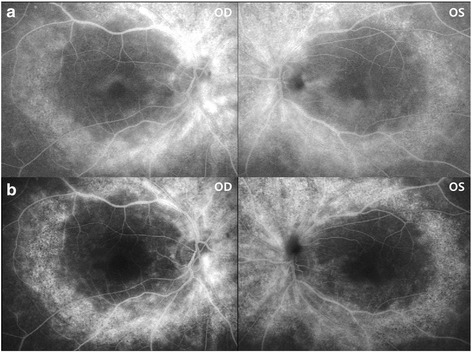
Fluorescein angiography (FA) images in the patient before (**a**) and after (**b**) oral acetazolamide therapy obtained at 2 min after fluorescein injection. Dye leakage in both the macular and mid-peripheral areas is decreased by the treatment, as demonstrated by remarkably decreased leakage in the macula and more definite demarcation of hyperfluorescent lesion in the mid-peripheral retina

During the patient’s visit of visual complaint, slit lamp examination showed no specific findings and there was no inflammation in the anterior chamber or vitreous cavity; however, her BCVA had declined to 20/50. SD-OCT examination showed the presence of a cystoid space within the inner retina and fluorescein angiography (FA) showed leakage in the macular area, a presentation typically associated with cystoid macular edema (CME), as well as diffuse leakage in the midperiphery (Figs. 2 and 3). Central macular thickness (CMT) was 245 μm and 335 μm in the right and left eyes, respectively. The patient was prescribed oral acetazolamide (250 mg) once a day.

After treatment with oral acetazolamide for one month, CME was resolved in both eyes on SD-OCT images (Fig. 2). CMT was decreased from 245 to 177 μm and from 335 to 146 μm in the right and left eyes, respectively. BCVA was improved to 20/25 in both eyes. Follow-up FA showed decreased dye leakage in both the macular and mid-peripheral areas (Fig. 3).

## Discussion

HCQ retinopathy presents with initial photoreceptor damage in a classic parafoveal distribution, known as a “bull’s eye” pattern, which corresponds to parafoveal scotomas upon visual field examination [8, 9] and parafoveal thinning of the outer nuclear layer with breakup of the ellipsoid zone and interdigitation zone lines on SD-OCT. [10] However, recent studies have shown that the initial pattern of damage in Asian eyes is more frequently in the more peripheral extramacular area near the arcades, as a pericentral pattern [11, 12]. In this pattern of retinopathy, CME may threaten relatively preserved central vision, leading to deterioration of visual function.

CME can develop in various retinal disorders [13]. Macular edema associated with chloroquine (CQ) retinopathy is relatively rare, and it was reported in 5 of 78 patients during a study period from 1957 to 1979 [14]; however, its treatment has not been discussed extensively. Although some cellular and molecular factors have been elucidated, the precise mechanisms for the formation of CME are unknown, including for HCQ retinopathy. In retinitis pigmentosa, CME formation has been suggested to involve breakdown of the blood-retinal barrier (BRB) as a result of chronic, low-grade inflammation [15–21] and decreased fluid transport efficiency of the retinal pigment epithelium [3]. CME associated with HCQ retinopathy has been reported in both leaking [22] and non-leaking [23] forms. In this case, it can be assumed that the leaking form of CME occurred owing to a mechanism such as BRB breakdown due to HCQ damage and that CME developed because such damage was not recovered even after the drug was cut off; however, the precise mechanism should be revealed in future studies.

In the present case, the CME associated with HCQ retinopathy exhibited diffuse leakage on FA that resolved with oral acetazolamide as evidenced by decreased fluorescein leakage. Acetazolamide reduces macular edema and improves visual acuity in some patients with macular edema related to certain inflammatory and degenerative eye diseases, including chronic iridocyclitis and retinitis pigmentosa [4]. With respect to mechanism, acetazolamide has been suggested to stimulate outward active transport and passive permeability across the BRB [6, 24, 25]. More specifically, acetazolamide blocks the active transport of certain ions (HCO3-, Cl-) across the retinal pigment epithelium [26], and also hastens the rate of resorption of subretinal fluid [27]. In our patient, acetazolamide was thought to induce functional recovery of the BRB, leading to a reduction in diffuse leakage and also improvement of CME.

Use of topical dorzolamide or oral acetazolamide (250 mg/day) in patients with CME in HCQ retinopathy has been described in only one recent study [1], which reported limited benefit. However, the patients in that study also had epiretinal membrane, which may have limited the beneficial effects of acetazolamide on reducing macular edema. Aside from carbonic anhydrase inhibitors, other treatment options for CME with HCQ retinopathy such as triamcinolone or anti-vascular endothelial growth factor antibodies have not been described in the literature. In our case, there were no accompanying structural alterations to the central macular area such as epiretinal membrane, which likely explains why acetazolamide was effective for anatomic and functional improvement of CME.

Retinitis pigmentosa associated with various types of mutations shows similar features to advanced HCQ retinopathy; therefore, retinitis pigmentosa should be carefully assessed for the differential diagnosis in patients taking HCQ medication. In the present case, the patient reported no family history of eye diseases and no visual symptoms before the initiation of HCQ therapy and she had no auditory symptoms. Genetic analyses on the associated mutations might be helpful for ruling out the possibilities of retinitis pigmentosa; however, we believe that the baseline (at the time of HCQ initiation) full-field electroretinography (ERG) and/or multifocal ERG may be very suggestive for the differential diagnosis.

## Conclusion

In conclusion, this case suggests that oral acetazolamide is an effective treatment for CME associated with HCQ retinopathy. Further prospective and comparative studies with a larger population are needed to assess the efficacy and safety of this treatment in patients with CME secondary to HCQ retinopathy.

## Abbreviations

BCVA: Best corrected visual acuity
BRB: Blood-retinal barrier
CME: Cystoid macular edema
CMT: Central macular thickness
CQ: Chloroquine
FA: Fluorescein angiography
HCQ: Hydroxychloroquine
RPE: Retinal pigment epithelium
SD-OCT: Spectral-domain optical coherence tomography
SLE: Systemic lupus erythematosus

## References

[1] Kellner S, Weinitz S, Farmand G, Kellner U (2014). Cystoid macular oedema and epiretinal membrane formation during progression of chloroquine retinopathy after drug cessation. Br J Ophthalmol.

[2] Grover S, Apushkin MA, Fishman GA (2006). Topical dorzolamide for the treatment of cystoid macular edema in patients with retinitis pigmentosa. Am J Ophthalmol.

[3] Cox SN, Hay E, Bird AC (1988). Treatment of chronic macular edema with acetazolamide. Arch Ophthalmol.

[4] Fishman GA, Gilbert LD, Fiscella RG, Kimura AE, Jampol LM (1989). Acetazolamide for treatment of chronic macular edema in retinitis pigmentosa. Arch Ophthalmol.

[5] Fishman GA, Gilbert LD, Anderson RJ, Marmor MF, Weleber RG, Viana MA (1994). Effect of methazolamide on chronic macular edema in patients with retinitis pigmentosa. Ophthalmology.

[6] Moldow B, Sander B, Larsen M, Engler C, Li B, Rosenberg T, Lund-Andersen H (1998). The effect of acetazolamide on passive and active transport of fluorescein across the blood-retina barrier in retinitis pigmentosa complicated by macular oedema. Graefes Arch Clin Exp Ophthalmol.

[7] Wolfensberger TJ (1999). The role of carbonic anhydrase inhibitors in the management of macular edema. Doc Ophthalmol.

[8] Marmor MF (2013). Efficient and effective screening for hydroxychloroquine toxicity. Am J Ophthalmol.

[9] Anderson C, Blaha GR, Marx JL (2011). Humphrey visual field findings in hydroxychloroquine toxicity. Eye (Lond).

[10] Brown DM, s Benz M, Wong TP, Major JC (2010). Spectral domain optical coherence tomography as an effective screening test for hydroxychloroquine retinopathy (the “flying saucer” sign). Clin Ophthalmol.

[11] Melles RB, Marmor MF (2015). Pericentral retinopathy and racial differences in hydroxychloroquine toxicity. Ophthalmology.

[12] Lee DH, Melles RB, Joe SG, Lee JY, Kim JG, Lee CK, Yoo B, Koo BS, Kim JT, Marmor MF (2015). Pericentral hydroxychloroquine retinopathy in Korean patients. Ophthalmology.

[13] Scholl S, Augustin A, Loewenstein A, Rizzo S, Kupperman B (2011). General pathophysiology of macular edema. Eur J Ophthalmol.

[14] Marks J (1982). Chloroquine retinopathy: is there a safe daily dose?. Ann Rheum Dis.

[15] Cunha-Vaz JG, Travassos A (1984). Breakdown of the blood-retinal barriers and cystoid macular edema. Surv Ophthalmol.

[16] Fishman GA, Cunha-Vaz J, Salzano T (1981). Vitreous fluorophotometry in patients with retinitis pigmentosa. Arch Ophthalmol.

[17] Kuchle M, Nguyen NX, Martus P, Freissler K, Schalnus R (1998). Aqueous flare in retinitis pigmentosa. Graefes Arch Clin Exp Ophthalmol.

[18] Mallick KS, Zeimer RC, Fishman GA, Blair NP, Anderson RJ (1984). Transport of fluorescein in the ocular posterior segment in retinitis pigmentosa. Arch Ophthalmol.

[19] Vinores SA, Kuchle M, Derevjanik NL, Henderer JD, Mahlow J, Green WR, Campochiaro PA (1995). Blood-retinal barrier breakdown in retinitis pigmentosa: light and electron microscopic immunolocalization. Histol Histopathol.

[20] Yoshida N, Ikeda Y, Notomi S, Ishikawa K, Murakami Y, Hisatomi T, Enaida H, Ishibashi T (2013). Laboratory evidence of sustained chronic inflammatory reaction in retinitis pigmentosa. Ophthalmology.

[21] Yoshida N, Ikeda Y, Notomi S, Ishikawa K, Murakami Y, Hisatomi T, Enaida H, Ishibashi T (2013). Clinical evidence of sustained chronic inflammatory reaction in retinitis pigmentosa. Ophthalmology.

[22] Bhavsar KV, Mukkamala LK, Freund KB (2015). Multimodal imaging in a severe case of hydroxychloroquine toxicity. Ophthalmic Surg Lasers Imaging Retina.

[23] Parikh VS, Modi YS, Au A, Ehlers JP, Srivastava SK, Schachat AP, Singh RP (2016). Nonleaking cystoid macular edema as a presentation of hydroxychloroquine retinal toxicity. Ophthalmology.

[24] Takahashi J, Mori F, Hikichi T, Yoshida A (2001). Effect of acetazolamide on outward permeability of blood-retina barrier using differential vitreous flyorophotometry. Curr Eye Res.

[25] Moldow B, Sander B, Larsen M, Lund–Andersen H (1999). Effects of acetazolamide on passive and active transport of fluorescein across the normal BRB. Invest Ophthalmol Vis Sci.

[26] Miller SS, Steinberg RH (1977). Active transport of ions across frog retinal pigment epithelium. Exp Eye Res.

[27] Marmor MF, Maack T (1982). Enhancement of retinal adhesion and subretinal fluid resorption by acetazolamide. Invest Ophthalmol Vis Sci.

